# Africa’s public health battle with cerebral malaria: What are we up against?

**DOI:** 10.5339/qmj.2024.55

**Published:** 2024-12-31

**Authors:** Abubaker Abdelmalik, Muataz Kashbour

**Affiliations:** 1 Faculty of Medicine, Misurata University, Misurata, Libya *Email: a.abdelmalik@med.misuratau.edu.ly; 2 Department of Radiology, National Cancer Institute, Misrata, Libya

**Keywords:** Malaria, cerebral malaria, Plasmodium falciparum, antimalarials, Sub-Saharan Africa, public health

## Abstract

Malaria continues to pose a significant public health challenge in Africa, where 95% of global malaria cases and deaths occur in Sub-Saharan Africa (SSA). Cerebral malaria (CM) is a fatal type of severe malaria primarily caused by *Plasmodium falciparum* species and disproportionately affects children under five years. Despite ongoing control efforts, CM persists as one of the most prevalent presentations of severe malaria with surprisingly high prevalences even in regions with lower endemicity and transmission rates. This commentary presents an updated compilation of key CM-related public health challenges, including delayed presentation of cases and a lack of diagnostic tools in resource-limited African communities, leading to misdiagnosis. Further challenges include insufficient provision of anti-malarial drugs and inaccessibility in rural and remote areas, coupled with emerging resistance to the gold-standard therapy (artemisinin combination therapy). CM carries a high risk of long-term neurological complications and is seen in almost half of the survivors. These complications severely impair their daily quality of life and extend to social and financial challenges. CM survivors also suffer from the lack of appropriate health services such as continuous rehabilitation, medical care, and psychological support. Despite its burden, interventional research in CM management remains insufficient, particularly regarding short- and long-term neurological complications. Local African governments have occupied the backseat for the length of this continental health crisis. It is crucial for them to occupy a proactive role in supporting healthcare infrastructure and delivering high-quality health services. Intragovernmental collaborations and establishing a regional health network within Africa can ensure realistic and accurate surveillance data tracking. From this, strategic healthcare planning, control measures, and source allocation could be better observed and tailored to the needs of different African subpopulations.

## 1. Background

Global reports on malaria estimated a worldwide prevalence of 247 million cases and 619 thousand deaths in 2022, the highest reported in a decade.^1^ Malaria has long been a global health issue accounting for 7.8% of the global disease burden, and is currently endemic in 84 countries.^1^ The African continent bears a major brunt of the infectious disease, with 29 Sub-Saharan countries being responsible for 95% (234 million) of worldwide cases and 96% (593 thousand) of total deaths. These figures have imposed a considerable strain on Africa’s healthcare system and threaten its already fragile public health services and facilities.

*Plasmodium* is the malaria-causing parasite, and of its five human-infecting species—*vivax, ovale, malariae, knowlesi,* and *falciparum*—the latter is significantly responsible for most morbidity and mortality and is mostly endemic in Sub-Saharan Africa (SSA).^2^ *Falciparum* species is the common cause of the fatal type of malaria known as severe malaria.

The overwhelming prevalence of malaria in Africa is multifaceted; various complex elements have made its control and mitigation difficult. Degarege and colleagues in their meta-analysis of 75 studies explored socioeconomic factors linked to malaria’s occurrence. Issues like lack of education, minimal income, substandard living conditions, and farming increased the risk of contracting malaria.^3^ However, De Silva and Marshall noted in their systematic review that malaria vectors conform to urbanization with transmission being concentrated around breeding sites, such as urban agriculture farms and low socioeconomic neighborhoods.^4^

## 2. Cerebral Malaria Epidemiology

Severe malaria cases are most prevalent among children under five years and account for 78.9% of all deaths in Africa.^5^ One of its most common variants is the life-threatening encephalopathy, cerebral malaria (CM), which is slowly taking preponderance over other variants. Reyburn et al. in their study of 1,984 severe malaria patients from 10 African hospitals found CM to predominate in areas of low transmissibility. In such areas, CM had an increased case-fatality rate of 13% as opposed to 7% found in high transmissibility areas where severe malarial anemia (SMA) predominates.^6^ Hence, CM incidence declines with higher transmission intensity and rises within low transmission areas. It is thought that CM occurrence is influenced by acquired immunological factors, as repeated exposure (such as in high-transmission areas) during infancy provides protection and adaptation, which later precludes its development around the typical peak age of five. Moreover, a Kenyan longitudinal study spanning 18 years observed an increase of CM-to-SMA ratio from 0.2 to 1.0.^7^ Another study conducted over two decades in Mozambique revealed a shift in severe malaria presentation over the years, with an increase in CM cases and a decline in anemia and respiratory distress.^8^ As efforts to control malaria increase and transmission rates decrease, the incidence of CM is projected to rise. Systematic reviews examining trends and patterns related to malaria syndromes, age groups, and transmission intensity support this projection.^9,10^

Despite efforts, reliable data on prevalence and incidence of CM remains lacking. Most updated statistics are provided by the World Health Organization’s (WHO) World Malaria Report, which relies on data collected through local health ministries and hospital admissions, yet no reporting of CM or severe malaria numbers is seen in their annual reports. Given that malaria is mostly prevalent in impoverished communities, case reporting is subject to improper data collection methods, biased reporting, and limited access to care.^3^ Consequently, CM cases are likely underreported.

## 3. Diagonostic Challenges

Using light microscopy to visualize plasmodium species in blood smears has long been the mainstay of malaria diagnosis. Such testing is often unavailable in many regions of Africa as it requires substantial expertise, training, and laboratory facilities.^11^ On the other hand, rapid diagnostic tests (RDTs) that detect Plasmodium antigens have become widely adopted in Africa, owing to their light and quick use without the need for electricity or a laboratory setting.^11^ RDTs have made malaria diagnosis more attainable in Africa, yet presumptive clinical diagnosis based on pyrexia remains pervasive, especially in regions of diagnostic tool scarcity.

Diagnosing CM with certainty in Africa, even within tertiary healthcare settings, poses challenges. It requires fundoscopic examination for malaria retinopathy (a pathognomonic feature), lumbar puncture, and brain imaging.^5^ In the face of restricted medical resources, many healthcare providers resort to circumstantial diagnosis based on the merit of febrile comatose presentation. Unfortunately, this approach contributes significantly to incorrect CM diagnosis, as post-mortem autopsies have shown that about 25% of clinically diagnosed cases are misdiagnosed.^5^ Moreover, it is essential to recognize that coma with convulsions in African patients may be attributed to other variants of severe malaria and not frank CM, such as hypoglycemia, metabolic acidosis, shock, or another non-malarial infectious source.

## 4. Treatment Challenges

### 4.1. Delayed presentation

A contributing challenge to the burden of CM in African nations is the delayed presentation of cases. Borgstein et al.^12^ in their 20-year-long analysis of 1,663 Malawian children with CM revealed that an alarming 57% of patients presented late. This also means a longer duration of coma and, therefore, higher mortality and devastating neurological sequelae. Delayed presentation to appropriate healthcare facilities was attributed to referral and institutional delays in 54% of cases. While 19% and 8% were due to a lack of caregiver prompt action and transportation issues, respectively.^12^ Other studies have attributed the delayed presentation to economic status, lack of transportation, and household dynamics.^13^ Hence, implementing structured and timely referral policies, providing education to caregivers, and providing access to transportation would be great strategies to mitigate the burden of CM in Africa.

### 4.2. Antimalarials

CM is a medical emergency with high mortality occurring within a few hours following presentation; thus, immediate resuscitation and prompt initiation of parenteral antimalarial drugs are imperative. Several clinical trials have shown superior efficacy of parenteral artesunate over parenteral quinine (the only two available parenteral antimalarial classes) in reducing mortality among both adults and children.^14^ The WHO also recommends the use of parenteral artesunate over quinine for the first 24 hours of presentation and until oral medication is tolerated, at which transition to oral therapy using artemisinin-based combination therapy (ACT) may commence.^15^

Artemisinin combination regimen is based on derivatives of artemisinin coupled with another one or two antibiotics. It remains the first-line therapy given the rampant resistance of *P. falciparum* to chloroquine in Africa. The oral use of artemisinin monotherapy is not recommended due to resistance susceptibility and shorter half-life. Emerging resistance to ACT resistance has begun to surface in some regions of SSA, notably in Uganda, Rwanda, and Eritrea.^16^ In the current state, ACT remains very effective; however, progression of resistance is of paramount concern. Appropriate mapping and surveillance of the extent of resistance and genotypic chasing of mutations are required to sustain treatment efficacy.

Another contributing factor to sustained malarial infestation concerning pharmacological intervention is insufficient drug coverage. Shortages of anti-malarial drug provision and supply chains have been reported, especially in rural areas and out-of-reach communities. This is attributed to funding delays coupled with flawed procurement logistics and inappropriate utilization by healthcare facilities.^17^ High medication costs and overtake of private sectors and unofficial drug retail have perpetuated treatment paucity. In addition, there is a concerning abundance of falsified antimalarial drugs; a drug quality study carried out in Africa and Asia revealed that 36% of antimalarial drugs were counterfeited, and as much as 43% of ACT.^18^

**Figure 1:**
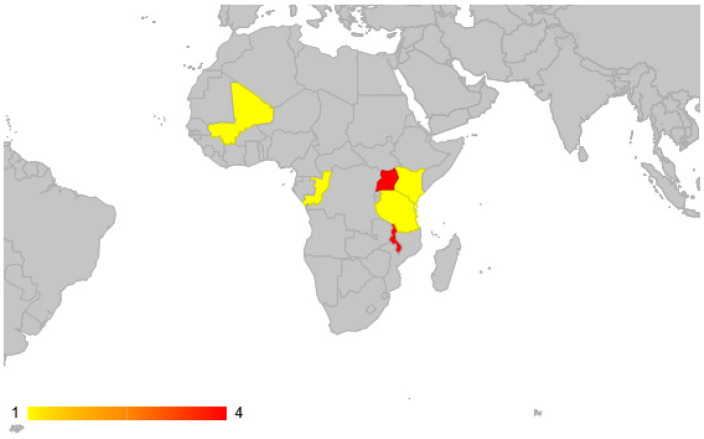
Quantitative and geographical distribution of clinical trials conducted on cerebral malaria across Africa. Malaria is endemic in 29 African countries, only twelve clinical trials on cerebral malaria were conducted and are registered on the National Health Institute clinical trials database (clinicaltrial.gov).^19^ Malawi and Uganda have the highest quantity of studies (four trials). The remainder of the countries highlighted in yellow have a single trial conducted.

In addition to antimalarial therapy, CM patients often require airway management, anticonvulsants, antipyretics, and other supportive measures. However, research on tailored management outcomes for CM patients remains limited. Only 12 registered trials in the African region have explored CM treatments, with just seven concluded studies, and only four were conducted in high malaria burden areas (Figure 1).^19^ Evidently, CM is an underfunded and overlooked area of research that needs exploring of management outcomes especially for interventions related to short- and long-term neurological implications.

## 5. Long-Lasting Neurological Challenges

CM carries the gravest long-term sequelae of all severe malaria variants, affecting 35–50% of survivors with some form of residual neurological impairment. Ranging from functional disability to cognitive and learning deficits to movement disorders.^20^ This sentences survivors and their caregivers to lifelong challenges, necessitating medical, financial, and psychosocial support. Given the socioeconomic disadvantage of many African families, providing long-term rehabilitation or specialized education services is almost unattainable. Stigmatization and isolation within communities add to the burden, especially when conditions like seizures and epilepsy are perceived as sorcerous in African cultures.^21^ Moreover, families experience burnout from continuous care, putting children at risk of abuse and neglect. As measures to decrease malaria mortality progress, the number of disabled CM survivors is expected to rise.

## 6. Imapct of COVID-19 on Disease Burden

African countries have historically suffered from broken and inadequate healthcare systems and services. This was recently evident during the COVID-19 pandemic when a moderate health-service disruption caused an upsurge in malaria cases and deaths.^1^ From 2019 to 2020, Africa witnessed an additional 15 million cases and 68 thousand deaths. The pandemic imposed a strain on the African health system as services were diverted to the pandemic at the cost of services tailored to malaria, leading to the inflation of CM cases.

## 7. Prevention and Control Challenges

Strategies for malaria control have been set in place by the global malaria action plan (GAMP) and the WHO in 2008 in an aim to protect humans and impose effective mosquito control.^1,15^ Current human protection measures include preventing mosquito bites, prophylactic pharmacotherapy, and vaccination. The administration of preventative antimalarial drugs to children in either seasonal or year-round transmission areas has proven effectiveness in reducing morbidity and mortality with a 30% reduction in incidence in SSA as reported by Esu et al. in their Cochrane review.^22^

Vaccination is also another promising prevention and control method with two WHO-approved vaccines currently in use for children. The RTS, S/ASO1 and the R21/Matrix-M vaccines are both derived from *P. falciparum* antigens and have been implemented in many African countries’ national immunization programs and in Sub-Saharan areas of moderate to high transmission. Both vaccines showed 70–77% efficacy in phase III trials; however, they lack a significant effectiveness against severe malaria.^23,24^ Moreover, concrete data regarding the longevity of their protection remain questionable.

Host protection using insecticide-treated bed nets (ITNs) is associated with a 17% reduction in children all-causes mortality and a 44% decrease in the incidence of severe malaria as reported by a 2018 systematic review of 23 clinical trials.^25^ The insecticide pyrethroid is the most used formulation in ITNs; however, mosquito resistance in many SSA has emerged (mainly West Africa). This has led to the shift to multi-formulated ITNs; WHO recommends the chlorfenpayr-pyrethroid formulation over pyrethroid-only ITNs.^15^ In addition to ITNs, indoor residual spraying (IRS) of walls and ceilings with insecticides, like pyrethroid, is also a recommended strategy by GAMP and WHO.^15^

Eliminating breeding sites and larval control is considered an effective supplementary strategy yet poses the effortful challenge of locating all individual vector breeding nests.^15^ This is done by physical removal of breeding sites or application of larvicides. Moreover, alteration of the vector environment by natural larva eaters and larvivorous fish species has also been employed.^26^ Such strategies are difficult to scale up due to the requirement of a large workforce for physical removal and the emergence of larvicide resistance. Moreover, the larvivorous fish tends to survive in certain local water conditions making it impossible to import or breed into different water conditions.^26^

## 8. Limited Government Backing

African officials must engage more in developing strategies and enforcing actions against malaria. It is reported that only 11% of funds against malaria are sourced from national governments, while 89% are funded internationally.^27^ Most African governments tackle malaria only through the Ministry of Health rather than a national or regional collaborative duty. For instance, China’s intragovernmental and multi-sectoral efforts involving 13 ministries helped reduce the disease rate and achieve a disease-free state in 2021.^28^ Therefore, African governments cannot rely solely on health ministries for effective control.

## 9. Conclusion

CM is spreading in regions with previously low incidence rates, and reducing its burden is challenging due to inaccessible diagnostic methods, late case presentations, and shortages in anti-malarial drugs. The situation is worsened by the high costs and prevalence of counterfeit medications. Emerging partial resistance is ominous and needs to be addressed promptly. CM survivors often endure persistent neurological damage, impacting their daily lives and places a strain on families and healthcare systems. There is a critical need for more research and data on CM treatment and its neurological outcomes. These challenges need to be well communicated and addressed in an efficient strategic manner with African authorities on the frontlines of the matter, or else risk losing the battle against this form of malaria.

## Authors’ Contribution

All authors participated in the collection, review, and analysis of the literature equally. Likewise, the rough writing, review, and final editing of the manuscript were mutually contributed to by all authors.

## Conflict of Interest Statement

The authors have no conflict of interest to declare.
